# Hydrastis Canadensis in Uterine Hæmorrhage, and Menorrhagia ; and Also in Dysmenorrhea

**Published:** 1877-08

**Authors:** W. A. Gordon

**Affiliations:** Hannibal, Mo.


### HYDRASTIS CANADENSIS IN UTERINE HÆMORRHAGE, AND MENORRHAGIA; AND ALSO IN DYSMENORRHEA.

By W. A. Gordon, M. D., Hannibal, Mo.

I have not seen *Hydrastis Canadensis* prominently spoken of in the leading text-books, as a reliable agent in hæmorrhage, from any of the mucous surfaces or in any respect worthy of especial notice further than as a good bitter tonic; and recommended in a general way as possessing merit in chronic diseases of the mucous membranes.

During the past ten years, I have made quite extensive use of hydrastis prepared in the form of tincture from the fresh root, with such positive and satisfactory results in uterine hæmorrhage, that I now seldom resort to any other remedy. The tincture I use is prepared after the following formula:

R. Rad. hydras. can. (fresh) - ozii.
Aq. dest. - - oj.

Maintain at a temperature of 120° F., for 24 hours; then add spts. rec. Oj—remove from the bath—and in three days it is ready for use. In those urgent cases where I formerly resorted to half-drachm and drachm doses of the fluid extract of ergot every twenty or thirty minutes, I now use the tincture of hydrastis in doses of from twenty to thirty drops, repeated the same as ergot, until the active hæmorrhage is controlled. The remedy is then continued in small doses—say two to five drops—every two to four hours, according to the urgency of the symptoms. In cases of marked prostration from loss of blood, I combine with the hydrastis, the tincture cinchonæ flavæ, in small doses, say from five to eight drops, which combination seems to produce a quiet and gradual contractility of both muscular fibres and capillary vessels of the womb; and effects a more natural and comfortable reaction from the extreme prostration attending this class of cases, together with an entire absence of cerebral and gastric disturbances which are so frequently concomitant symptoms following the administration of large doses of ergot.

In *Menorrhagia*, I have found it to give decided and prompt relief. In this class of cases, I am in the habit of giving from two to five drops of the above tincture of hydrastis in a teaspoonful of water every two or three hours, or oftener, and in larger doses if the urgency of the symptoms demands it. After the flow is brought to its normal quality, the minimum dose is continued twice a day until the next period of menstruation, when if the excessive discharge recurs—resort again to longer and more frequent doses, until it is brought under control.

In *Dysmenorrhœa* caused by chronic endo-metritis, the tincture of hydrastis with bromine has given me very satisfactory results.

In the use of bromine as an internal therapeutical agent, I have observed that persons of a nervous temperament are highly susceptible to its influence. I would here incidentally remark, that during our late war, I had occasion to use it quite extensively in the military hospitals of the Department of Kentucky, under the direction of Dr. M. Goldsmith, late surgeon U. S. V., and my observations there were such that in the majority of cases treated with bromine—which were hospital gangrene and erysipelas—the doses recommended and administered internally were not attended with as good results as much smaller doses, frequently repeated. See U. S. Dispensatory for preparation and dose.

In some cases, classified under the head *neuroses*, and especially those arising from diseased conditions of the procreative system, I have known one-twentieth the dose directed by the U. S. Dispensatory produce violent headache, ranging from the frontal sinus along the track of the longitudinal sinus down to the base of the brain, with a marked increase of pulse in volume and frequency. In one instance the pulse was increased fifteen beats per minute, which lasted about two hours before it began to decrease, and did not resume its normal beat until the expiration of seven hours, from the time the dose was administered. This case was a nervous female afflicted with endometritis.

In these nervous susceptible cases, I have met with some that could not tolerate over ten drops of the following solution, four times a day without stimulation and headache:

℞. Bromini, gt. j.
Aq. dest. oj. M.

If a much larger dose than the above is continued for several weeks it will almost positively produce *membranous dysmenorrhœa.*

The formula I am now using in several cases of endo-metritis is to take equal parts of the above aqueous solution of bromine and tincture hydrastis, and give fifteen to twenty drops of the mixture three times a day, and if restless at night give a dose at bed-time.

My reasons for being somewhat explicit on the internal administration of bromine is from the fact, that its potency has so limited its use, that comparatively few members of the profession have ever given it a fair trial.

But if given in small doses, such as suggested above, or even smaller, I am satisfied that it is a remedy of more than ordinary merit as an alterative and stimulant to the procreative system, and at no distant day will be found to possess great value as a remedy for increasing cell action in the nerve centres controling the sexual system of man.

