# Correction

**Published:** 1877-08

**Authors:** 


Correction.—Owing to a mistake in the manuscript the formula for preparing the tincture of Hydrastis Canad. (p. 142) is not quite correct, *it should read six ounces* of fresh root instead of two. W. A. G.

